# Unconventional surgery for thoracic esophageal rupture with empyema and mediastinitis: a case report and literature review

**DOI:** 10.1186/s13019-023-02208-2

**Published:** 2023-06-13

**Authors:** Yong-jun Deng, Huan-peng Liu, Jian-bin Zou

**Affiliations:** Department of Thoracic Surgery, The Affiliated Hospital of Yunnan University (The Second People’s Hospital of Yunnan Province, Yunnan Eye Hospital), Kunming, Yunnan Province 650021 P.R. China

**Keywords:** Esophageal rupture, Surgical treatment, High pressure gas, Esophagogastrostomy

## Abstract

Treatment of esophageal perforation or rupture is complicated and controversial, especially in advanced cases. In fact, it is generally accepted that this disease must be treated individually according to the location, causes and clinical features of rupture or perforation. A very rare case was admitted to our department, who was injured 5 days ago by high-pressure gas of a running air compressor and resulted in a long-term longitudinal rupture of the thoracic esophagus. Although the patient suffered from empyema and mediastinitis at the same time, and his condition was very serious, the debridement and desquamation of empyema were still implemented, followed by left thoracic esophagectomy and left neck approach esophagogastrostomy in the same period successfully. The patient got a good result finally.

## Introduction

Spontaneous esophageal rupture is a rare but serious disease with a high mortality rate [1]. The etiology of esophageal rupture can be divided into iatrogenic instrument operation, spontaneous esophageal perforation, foreign body swallowing injury, chest injury, surgical accidental injury, esophageal chemical burns, tumor causes and others. Surgical treatment is the preferred surgical treatment, which can thoroughly remove pleural and mediastinal pollutants, avoid the formation of empyema, repair rupture and promote healing. Esophagus rupture caused by high pressure gas is rare, which is easily to be misdiagnosed at first time, leading to treatment delay; therefore, the life-saving rate is still low. Empyema refers to bacteria invading the pleural cavity, producing purulent exudate and accumulating in the pleural cavity, which is a clinical disease with high morbidity and mortality and often treated by surgical. Mediastinitis is mainly a downward necrotizing mediastinitis caused by deep sternal wound infection, esophageal perforation or ENT infection [2]. Researchers had reported the treatment of thoracic esophageal rupture and with concomitant empyema and mediastinitis [3, 4], but esophagus rupture caused by high pressure gas with concomitant empyema and mediastinitis had never reported.

We report a case injured by high pressure gas of a running air compressor, who had a long size of longitudinal rupture of thoracic esophagus and concomitant empyema treated with left thoracic and left neck approach esophagogastrostomy in the same period. Expecting to provide some effective treatment references for esophagus rupture caused by high pressure gas with concomitant empyema and mediastinitis.

## Case presentation

The patient was a 41-year-old male worker with a main complaint of lefts empyema and fever without special history. Informed consent was obtained from the patients in advance. Five days ago, when he was operating an air compressor, the gas pipe of the running air compressor fell off and intubated his mouth accidentally. The patient suddenly developed acute chest pain and shortness of breath, and he was sent to the local municipal hospital on the same day. Thoracic subcutaneous and mediastinal emphysemas as well as left encapsulated effusion were observed on computed tomography scan, and then closed drainage of his left pleural cavity was performed via the 7th intercostal space on the same day. Although the left lung was dilated after placing the thoracic catheter 2 days later, bubbles and thick pleural effusion were still be observed in the closed thoracic drainage device, and the patient began to fever. Then the patient was transferred to the department of thoracic surgery of our hospital on the 5 th day.

After admission, the physical examination, Blood testing and biochemical examination were performed and the results were shown in Table 1.

**Table 1:** The results of physical examination, Blood testing and biochemical examination

Indexes	numerical value
Height	170 cm
Weight	72 kg
BP	120/72 mmHg
HR	106/min
respiratory rate	25/min
SpO2	95%
WBC	17.3 × 10^9^/L
CRP	26 mg/L
serum total protein	53 mg/dl
serum albumin	29 mg/dl

**Fig. 1:**
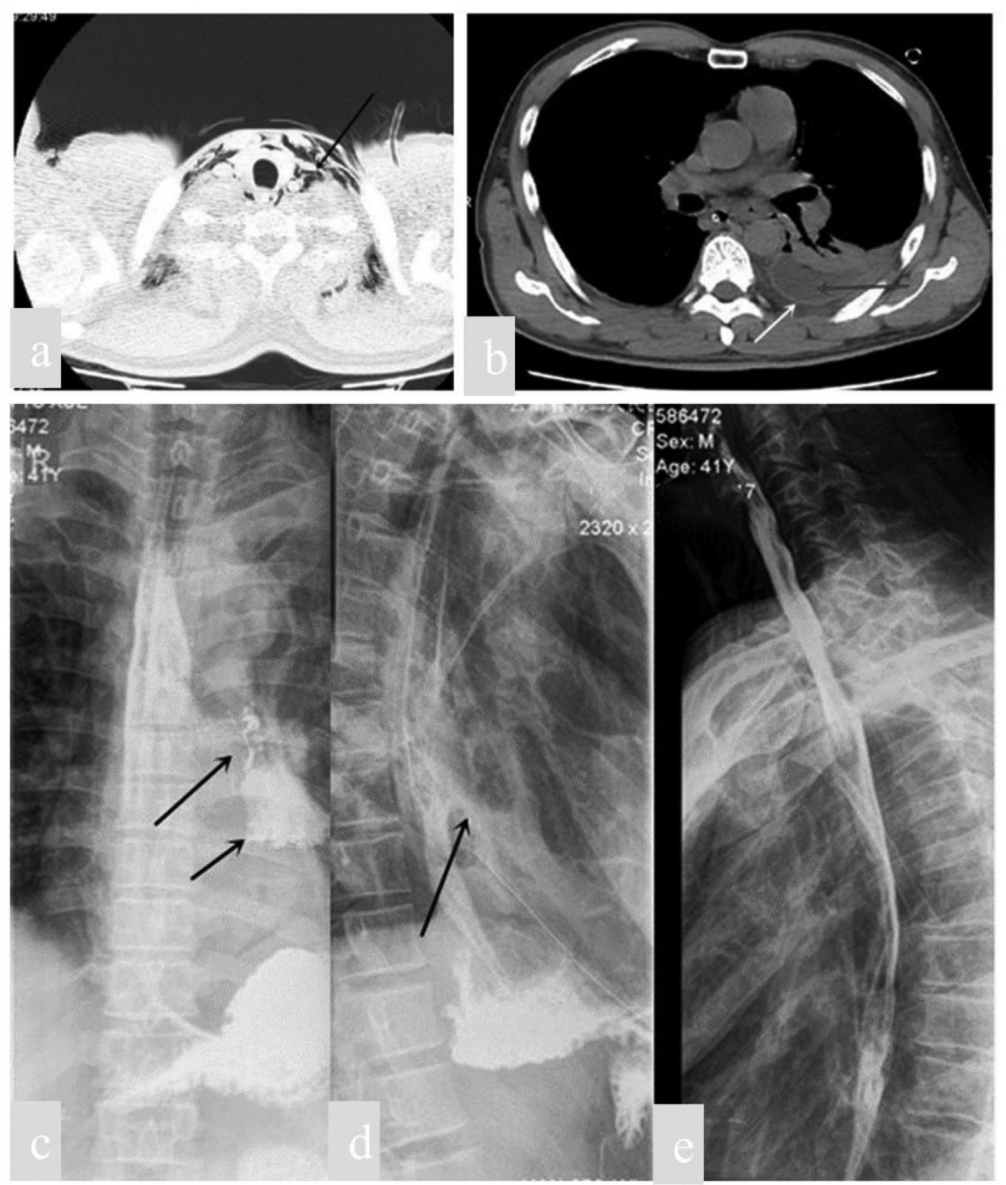
CT scan of the chest revealed subcutaneous and mediastinal emphysema in the chest and left Encapsulated Effusion. Esophagography showed an extensive tear on the left side of the middle and lower portions of thoracic esophagus. **a** Lung window, Arrow: subcutaneous and mediastinal emphysemas. **b** Mediastinal window, Arrow: encapsulated effusion. **c** Postero-anterior position, **d** left lateral position, **e** right lateral position. Arrow: contrast agent entered into left thoracic cavity through the ruptured esophagus

Esophagography showed an extensive tear on the left side of the middle and lower part of thoracic esophagus, and chest CT displayed pleural effusion (Fig. 1). Then the patient was diagnosed as a rupture of the thoracic esophagus and empyema. Surgery should be emergently performed in consideration of concomitant serious infection in the left thoracic cavity.

Initially, the patient was intubated with a double-lumen tube and then lying in a full right supine position. The left lung was deflated and one-lung ventilation was started. An 18 cm left posterolateral incision was made between the 6 th ribs. Pleural fiberboard thickening leads to chest wall adhesion.

A large amount of necrotic tissue, pus and residual food were found in the left thoracic cavity and mediastinum, which were surrounded by thickened viscera and parietal pleural fiberboard. A rupture site of about 7 cm was found in the middle and lower left side of the thoracic esophagus, and the rupture edge showed signs of necrosis. Then total thoracic esophagectomy and left cervical esophagogastrostomy were attempted simultaneously. Next, the necrotized substances were removed, empyema debridement and decortication were performed, followed by a large amount of massive irrigation. To facilitate operation and avoid pollution, the ruptured side of esophagus was sutured intermittently. Mobilization of the entire thoracic esophagus was performed from the level of diaphragmatic reflection to the thoracic inlet, and then the whole thoracic esophagus was resected, followed by massive irrigation with normal saline and Poseidon iodine repeatedly. Then the diaphragm was cut open and entered the abdominal cavity until the upper abdomen reached the best exposure state. Generally, the stomach was fully active at the small and large curvature of the cardia and stomach. A tubular stomach was constructed by resecting the lesser curvature of the stomach and cardia along a line about 4–5 cm from the edge of the greater curvature using a linear cutting stapler (Covidien, America), while a left cervical incision was made on the anterior edge of the sternocleidomastoid muscle and the cervical esophagus was moved. The tubular stomach was pulled up to the left neck along the esophageal bed, then a left cervical anastomosis was subsequently carried out in an end-to-side fashion close to the greater curvature of the tubular stomach using a 25 mm circular stapler (Covidien, America). The diaphragm was sutured and a 32 F chest tube was placed via the 7th intercostal space.

**Fig. 2:**
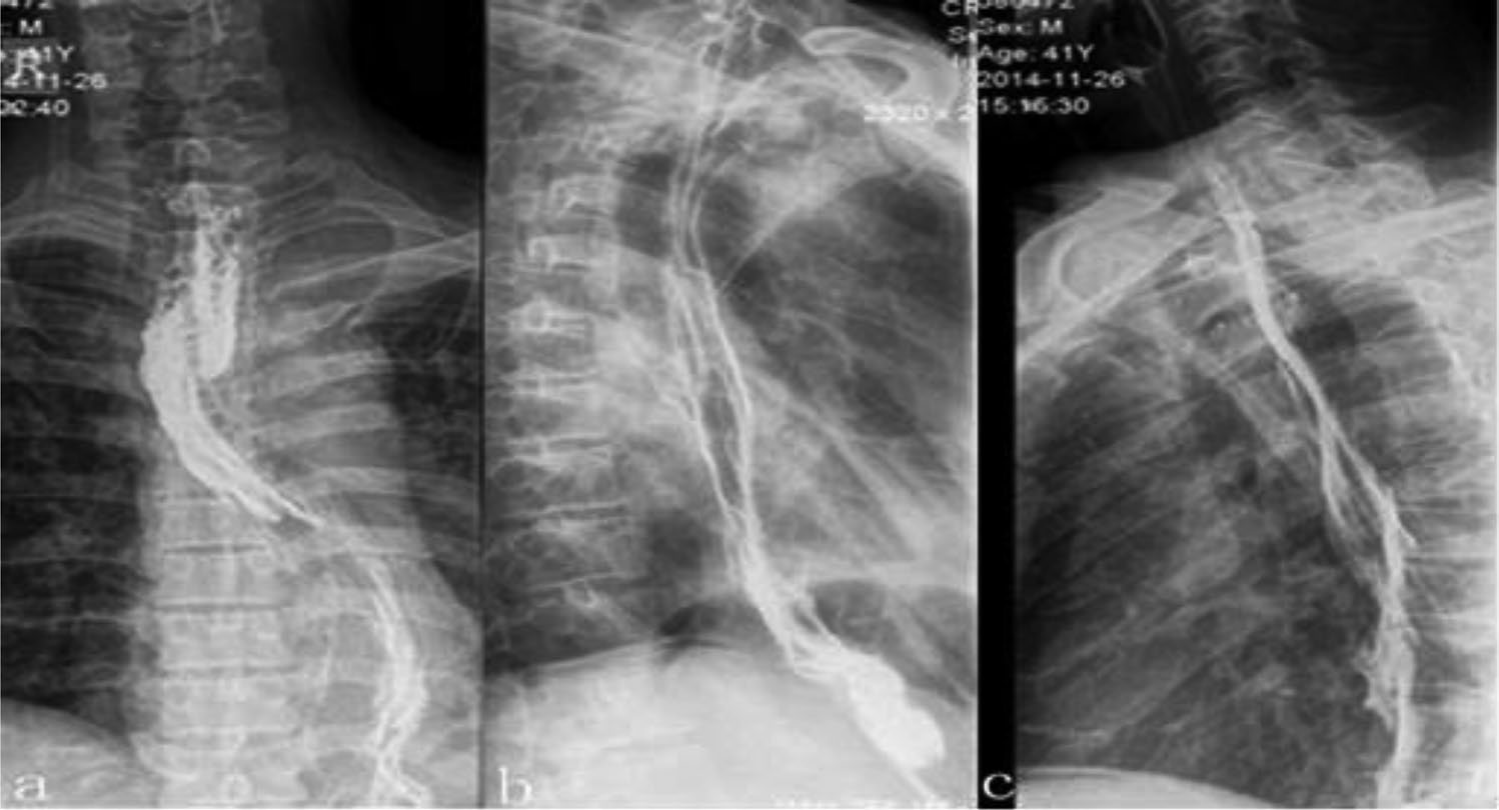
A postoperative esophagography showed no evidence of anastomotic leak. **a** Postero-anterior position, **b** left lateral position, **c** right lateral position

The overall postoperative performance of the patients was good. An esophagography using meglumine diatrizoate was performed on the 7th postoperative day, which showed no evidence of anastomotic leak (Fig. 2). The patient was discharged home on the 11th day after operation. Three months after the operation, the patient developed a symptom of dysphagia, and an anastomotic stenosis was confirmed which was relieved by endoscopic esophageal dilatation.

## Discussion

It has been reported that the most common cause of esophageal rupture or perforation is severe vomiting, accounting for 64%, followed by other mechanical factors, such as trauma, overeating and cough, accounting for 19% [5]. Traumatic esophageal ruptures accounted for 4–14% of all esophageal perforations [6]. Both traumatic esophageal rupture and spontaneous esophageal perforation had a low incidence and were associated with significant mortality. Early diagnosis and appropriate intervention were needed to reduce incidence rate and mortality [6, 7].

The awareness of this disease and doctor’s experience can make the early diagnosis of this disease. Actually, only about 30% of cases were diagnosed as rupture of the esophagus at their first visit to hospital. In case of doubt, esophagography should be performed with an aqueous contrast agent (such as meglumine diatrizoate). When mediastinal or thoracic leakage is observed, the disease can be diagnosed. CT may also be useful in the diagnosis of diseases, especially in these critically ill patients. Endoscopic examination seems to be a relative contraindication owing to need to inject air into the esophagus.

For treatment, primary repair can be performed in patients with esophageal perforation or rupture within 24 h. However, the treatment of late esophagus perforation or rupture remains controversial [8]. At present there is no consensus on the optimal treatment strategy to handle these patients due to the lack of many randomized controlled trials. Surgery and conservative treatment including thoracic cavity drainage are feasible options, but surgical treatment is safer and better than conservative procedures, and may achieve better results than conservative treatment [9–11]. Fukushima et al., reported that the mortality rates after surgery and conservative treatment were 7.7% and 50.0%, respectively, indicating that the prognosis of the surgical treatment group was good [12]. If surgical treatment is indicated, what is the choice of primary repair or esophagectomy with or without immediate reconstruction? The optimal treatment option is primary surgical repair of the rupture, removal of mediastinal thoracic contaminants, and removal of distal obstructive lesions. In fact, it is generally accepted that the treatment of this disease must be individualized according to the location, cause and clinical characteristics of the rupture or perforation (such as the length of time between onset and treatment, the degree of mediastinitis or chest infection, the extent of esophageal injury, concurrent medical conditions and hemodynamic stability) [13]. However, for patients with thoracic esophageal rupture accompanied by severe empyema and mediastinal inflammation, surgery may be difficult and dangerous. Moreover, as far as we know, there is no such report.

The patient not only had a large esophageal rupture, but also had a time interval of more than 24 h between the occurrence and diagnosis of the rupture. Was it reasonable for the patient to perform thoracic esophagectomy and cervical esophagogastrostomy at the same period under the condition of serious pleural infection? It has been recommended that esophagectomy should be conducted if extensive esophageal rupture occurs [9], and the length of esophageal rupture was considered as an indicator of esophagectomy [14]. Okonta KE suggested that esophagectomy was superior to conservative treatment for delayed benign esophageal perforation (defined as a perforation diagnosed after 24 h) [10]. Although the left thorax of the patient was seriously infected, his nutritional status was good and his hemodynamics was relatively stable. After thoracotomy, the pus and necrotic tissue in the thorax could be completely removed. After repeated large-scale washing with normal saline and Poseidon iodine, the thorax was very clean, almost reaching the level of wound cleaning. So we performed an entire thoracic portion esophagectomy with an immediate reconstruction with left neck approach esophagogastrostomy according to our experience, furthermore the tubular stomach was pulled up to the left neck trans-esophageal bed way, rather than trans-substernal pathway. Because of the simultaneous anastomosis, the patient avoided esophageal rejection and staged surgery, and achieved good results.

## Conclusion

Esophageal rupture caused by high-pressure gas is rare, and surgery is an effective treatment option for such disease. It was safe and technically feasible for our treatment strategy to undertake an esophagectomy with an immediate reconstruction with left neck approach esophagogastrostomy for the patient with an extensive thoracic esophageal rupture and concomitant empyema and mediastinitis.

## Data Availability

All data, models, and code generated or used during the study appear in the submitted article.
